# Mechanisms of Action in FLASH Radiotherapy: A Comprehensive Review of Physicochemical and Biological Processes on Cancerous and Normal Cells

**DOI:** 10.3390/cells13100835

**Published:** 2024-05-14

**Authors:** James C. L. Chow, Harry E. Ruda

**Affiliations:** 1 Radiation Medicine Program, Princess Margaret Cancer Centre, University Health Network, Toronto, ON M5G 1X6, Canada; 2 Department of Radiation Oncology, University of Toronto, Toronto, ON M5T 1P5, Canada; 3 Centre of Advance Nanotechnology, Faculty of Applied Science and Engineering, University of Toronto, Toronto, ON M5S 3E4, Canada; harry.ruda@utoronto.ca; 4 Department of Materials Science and Engineering, University of Toronto, Toronto, ON M5S 3E4, Canada

**Keywords:** FLASH, normal cell sparing, cancer cell kill, cell function, radiotherapy, ultra-high dose rate, preclinical model, oxygen depletion, biological process, physicochemical process

## Abstract

The advent of FLASH radiotherapy (FLASH-RT) has brought forth a paradigm shift in cancer treatment, showcasing remarkable normal cell sparing effects with ultra-high dose rates (>40 Gy/s). This review delves into the multifaceted mechanisms underpinning the efficacy of FLASH effect, examining both physicochemical and biological hypotheses in cell biophysics. The physicochemical process encompasses oxygen depletion, reactive oxygen species, and free radical recombination. In parallel, the biological process explores the FLASH effect on the immune system and on blood vessels in treatment sites such as the brain, lung, gastrointestinal tract, skin, and subcutaneous tissue. This review investigated the selective targeting of cancer cells and the modulation of the tumor microenvironment through FLASH-RT. Examining these mechanisms, we explore the implications and challenges of integrating FLASH-RT into cancer treatment. The potential to spare normal cells, boost the immune response, and modify the tumor vasculature offers new therapeutic strategies. Despite progress in understanding FLASH-RT, this review highlights knowledge gaps, emphasizing the need for further research to optimize its clinical applications. The synthesis of physicochemical and biological insights serves as a comprehensive resource for cell biology, molecular biology, and biophysics researchers and clinicians navigating the evolution of FLASH-RT in cancer therapy.

## 1. Introduction

Radiotherapy (RT), extensively utilized in cancer treatment, utilizes high-energy ionizing radiation such as X-rays, electrons or protons to specifically target and disrupt cancer cell reproduction by inducing damage to its DNA. This approach inhibits the growth and division of cancer cells effectively [1,2,3]. While RT is a powerful method for treating various cancers, its drawback lies in potential damage to healthy cells, limiting the radiation dosage administered to tumors [4,5]. This constraint often results in incomplete tumor eradication and diminishes overall treatment efficacy [6]. To address these challenges, there is ongoing research to optimize RT outcomes based on cell radiobiology. Current techniques, such as stereotactic body RT [7] and intensity-modulated RT [8], aim to enhance targeted radiation to tumors while minimizing exposure to surrounding healthy tissues or cells. Despite these advancements, conventional radiotherapy (CONV-RT) still requires multiple sessions [9], spanning weeks, and necessitates patient travel to cancer centers. This extended treatment duration can pose an additional burden on patients and their families.

FLASH radiotherapy (FLASH-RT) presents an innovative approach to traditional RT by leveraging ultra-high dose rates (UHDR) to address the challenges associated with radiation-induced toxicity [10]. While CONV-RT relies on ionizing radiation to damage and eliminate cancer cells, the potential harm to surrounding healthy cells imposes limitations on the administered dosage [11]. FLASH-RT, characterized by a delivery rate several orders of magnitude higher than conventional clinical RT, introduces the FLASH effect, involving UHDR of greater than 40 Gy/s [12,13,14]. This unique characteristic significantly reduces damage to healthy cells while preserving the potent antitumor effectiveness of the treatment. Although the concept of FLASH-RT was initially introduced by Dewey and Boag in 1959 [15], it did not gain notable attention until after 2014 by Favaudon et al. [16] when in vivo studies demonstrated its ability to minimize normal cell toxicity while achieving comparable tumor control to CONV-RT [17,18,19].

Studies on FLASH-RT using ion beam RT are currently an area of active investigation, holding promise for significant advancements in cancer treatment. Ion beam therapy, known for its precise targeting of tumors while sparing surrounding healthy tissue, is being explored in combination with FLASH techniques to further enhance treatment outcomes. The application of UHDRs in ion beam FLASH-RT has the potential to exploit the unique physical properties of charged particles, such as protons and carbon ions [20], to deliver radiation with unprecedented speed and efficacy. Despite its nascent stage, preliminary preclinical studies have demonstrated encouraging results, highlighting the feasibility and potential benefits of FLASH-RT in ion beam therapy [21]. Nevertheless, additional research is required to gain a thorough understanding of the FLASH effect from both cell biology and biophysics perspectives and to refine treatment protocols for clinical application. Consequently, ongoing studies on FLASH-RT for ion beam therapy stand at the forefront of radiation oncology research, presenting promising opportunities for enhanced cancer management [22].

The advantages of FLASH-RT are evident in its potential to overcome the limitations posed by traditional RT. By minimizing radiation-induced injuries to healthy tissues, it reduces the treatment time and internal organ motion during irradiation [19]. FLASH-RT enables the delivery of higher radiation doses to tumors, enhancing treatment efficacy and potentially leading to more comprehensive tumor eradication. This innovative approach holds promise in transforming the RT, offering a solution to the challenges of conventional treatments and providing new avenues for improving patient outcomes in cancer care [20,21,22]. Table 1 provides a comprehensive comparison between FLASH-RT and CONV-RT across various aspects. FLASH-RT exhibits ultra-fast treatment times in milliseconds compared to the typical seconds to minutes seen in CONV-RT. Dose rates in FLASH-RT are extremely high, surpassing 40 Gy/s, while CONV-RT typically ranges from 0.001 to 0.4 Gy/s. Moreover, FLASH-RT demonstrates enhanced normal cell sparing due to its UHDR, contrasting with CONV-RT, which poses a greater risk to normal cells. The therapeutic index increased in FLASH-RT, while CONV-RT follows standard radiobiological principles. Moreover, FLASH-RT allows for single or few fractions, whereas CONV-RT commonly involves multiple fractions. Patient comfort is improved with FLASH-RT due to reduced overall treatment time, whereas CONV-RT often involves longer treatment sessions. Furthermore, FLASH-RT potentially reduces machine wear and tear, integrates with advanced imaging, and minimizes organ motion during treatment. It may also increase patient throughput, although treatment duration may impact this aspect. While FLASH-RT is investigational with ongoing research in clinical trials, CONV-RT is an established and widely practiced treatment option. In terms of cost and accessibility, FLASH-RT may incur higher costs but offers potential benefits in accessibility compared to CONV-RT.

It should be noted that FLASH-RT is an emerging technology and ongoing research, in particular cell radiobiology, is vital to validate its clinical benefits and address challenges. A key challenge in its clinical translation is understanding the intricate mechanisms of cell function, response, and killing in FLASH-RT. Unraveling the molecular and cellular processes that contribute to the unique FLASH effect is essential for optimizing treatment protocols, enhancing therapeutic outcomes, and minimizing potential side effects [23]. However, the challenge lies in the difficulty of conducting experiments to comprehensively understand the FLASH effect [24,25]. The UHDRs associated with FLASH-RT demand specialized equipment and sophisticated techniques that are not readily available in standard experimental setups such as the UHDR radiation sources [19,26]. Moreover, the rapid nature of FLASH radiation delivery poses challenges in capturing real-time cellular responses, making it intricate to dissect the precise mechanisms involved [27]. Despite these challenges, gaining a profound understanding of the cell-killing and cell-sparing mechanisms associated with FLASH-RT is crucial for advancing its clinical application, guiding treatment planning, and ultimately improving the overall efficacy and safety of cancer radiotherapy. Collaborative efforts between researchers in cell biology and biophysics, clinicians, and technological advancements will be instrumental in overcoming these experimental hurdles and unlocking the full potential of FLASH-RT in the pursuit of more effective and targeted cancer treatments.

In the rapidly evolving realm of cancer treatment, FLASH-RT has emerged as a promising avenue with the potential to revolutionize CONV-RT methods. However, despite its growing popularity, our understanding of the underlying mechanisms driving its efficacy remains incomplete [28,29,30,31]. This is where our comprehensive review is needed to fill a crucial gap of incomplete understanding of the underlying mechanisms driving the efficacy of FLASH-RT. By meticulously examining the physicochemical and biological processes involved in FLASH-RT, we aim to provide a holistic understanding of its mechanism of action based on cell biology and biophysics. Through this review, we not only synthesize the latest research findings but also offer insights into the direction of future investigations. This paper serves as an indispensable resource for researchers, clinicians, and stakeholders invested in advancing FLASH-RT as a cutting-edge cancer treatment modality. This review paper aims to examine the mechanisms of the FLASH effect in FLASH-RT focusing on the impact of cell function and response. Our objectives include providing a concise overview of the current understanding of the FLASH effect, identifying gaps in proposed mechanisms, and suggesting a roadmap for future research.

## 2. Mechanisms of the FLASH Effect in RT

**Figure 1:**
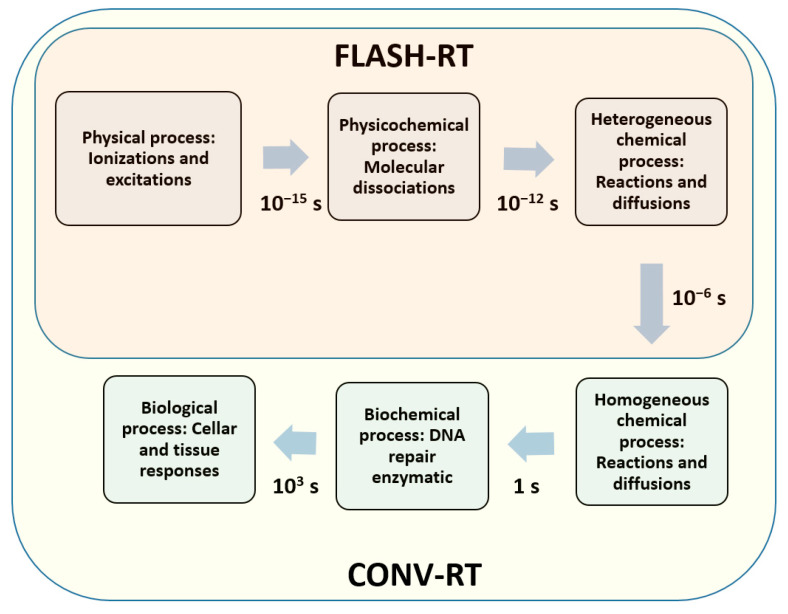
Schematic diagram showing the primary physicochemical and biological reactions subsequent to cellular and tissue exposure to radiation. CONV-RT disturbs both chemical and biological reactions, whereas FLASH-RT circumvents engagement with biochemical pathways, allowing it to bypass these reactions.

FLASH-RT is an innovative approach in cancer treatment that delivers an ultra-high dose of radiation in an extremely short duration, typically in milliseconds, as opposed to the CONV-RT administered over several minutes. The unique aspect of FLASH-RT lies in its ability to induce rapid cancer cell kill with reduced damage to surrounding healthy cells compared to traditional radiotherapy. The mechanism of cell kill in FLASH-RT is multifaceted and can be broadly categorized into physicochemistry and biology [32,33,34]. The physicochemical process is characterized by the rapid delivery of radiation, leading to a phenomenon where biological tissues absorb energy at an accelerated pace. The effect then involves the generation of reactive oxygen species (ROS) during radiation, impacting cellular components. On the other hand, the biological process encompasses the intricate interplay between the rapid radiation delivery and the cellular response, affecting DNA repair mechanisms and signaling pathways. When comparing the timelines of physicochemical processes in FLASH and CONV-RT, it is essential to note that FLASH irradiation is about 1000 times faster than conventional irradiation, as shown in Figure 1.

Understanding these interconnected aspects is important for optimizing FLASH-RT’s therapeutic potential while minimizing adverse effects on normal cells.

**Figure 2:**
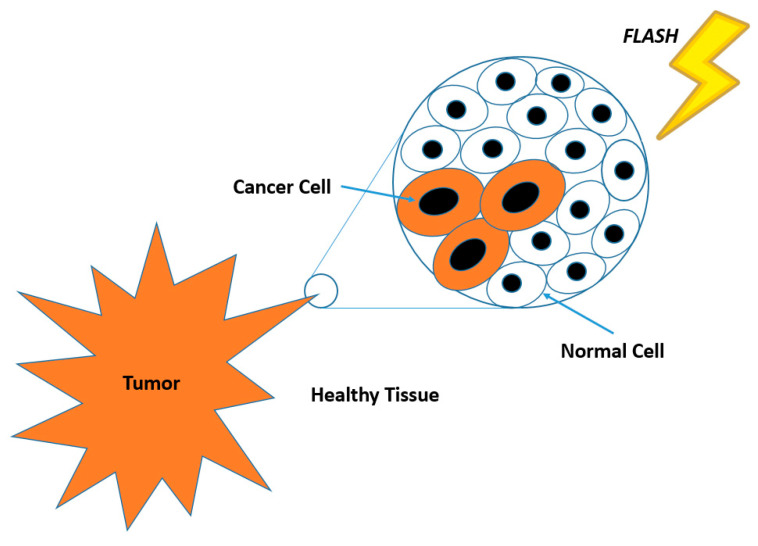
Schematic diagram showing the microenvironment when cancer cells have infiltrated the healthy tissue containing normal cells at the tumor margin.

When a tumor is excised prior to radiotherapy, the irradiated volume encompasses an added margin around the tumor or surgical site. This expansion aims to target tumor cells that may have infiltrated the surrounding healthy tissue. These infiltrated cancer cells occupy a distinct environment from those within the primary tumor, as shown in Figure 2. FLASH-RT offers the advantage of safeguarding healthy tissue while maintaining efficacy comparable to CONV-RT in combating cancer cells. The hypothesis regarding the effects of FLASH-RT suggests that the unique biological and physiological responses to UHDR irradiation in FLASH-RT contribute to its ability to target infiltrated cancer cells within normal tissues while minimizing damage to healthy surrounding tissue. One potential explanation for the effectiveness of FLASH-RT on infiltrated cancer cells lies in the differential response of normal tissue vasculature and tumor vasculature to UHDR irradiation. It is hypothesized that the rapid delivery of radiation may overwhelm the repair mechanisms of tumor vasculature while sparing the normal tissue vasculature. This could result in preferential damage to the tumor microenvironment while preserving surrounding healthy tissue. Moreover, the oxygenation status of tissues during FLASH-RT could play a role in its efficacy. Unlike CONV-RT, where temporary hypoxia in the tumor microenvironment can reduce treatment effectiveness, the rapid delivery of FLASH-RT may mitigate the impact of transient hypoxia. This could result in more consistent and effective tumor cell killing, even in regions with poor oxygenation.

### 2.1. Physicochemical Process on Cell Killing

The physicochemical process in FLASH-RT unfolds in distinct stages, primarily characterized by the interaction of energetic particles with water within femtoseconds. The distinction between UHDR and conventional dose rate lies in the duration of exposure, which significantly influences the initial radiation chemistry, as shown in Figure 1 [14]. During the physical stage, water molecules undergo electronic excitation and ionization, resulting in the formation of highly unstable ionized and excited molecules [35,36]. This stage, occurring between 10^−15^ and 10^−12^ s, involves de-excitation events such as proton transfer, dissociation, and electron thermalization. Proton transfer leads to the production of hydroxyl radicals, crucial in subsequent reactions [37]. The emitted electrons either migrate to form secondary ionizations or become thermalized, generating aqueous electrons. The nonhomogeneous chemical stage follows between 10^−12^ and 10^−6^ s, where radical species diffuse, react, and form new radical products [38]. In this intricate process, the spatial distribution of ionizations, represented by linear energy transfer (LET), dictates the competition between recombination and diffusion [39]. Notably, in FLASH-RT, the ultra-high dose is delivered in milliseconds, potentially altering the competition dynamics between radical recombination and diffusion in the cell, providing insights into the distinctive radiobiological effects observed in FLASH effect.

**Figure 3:**
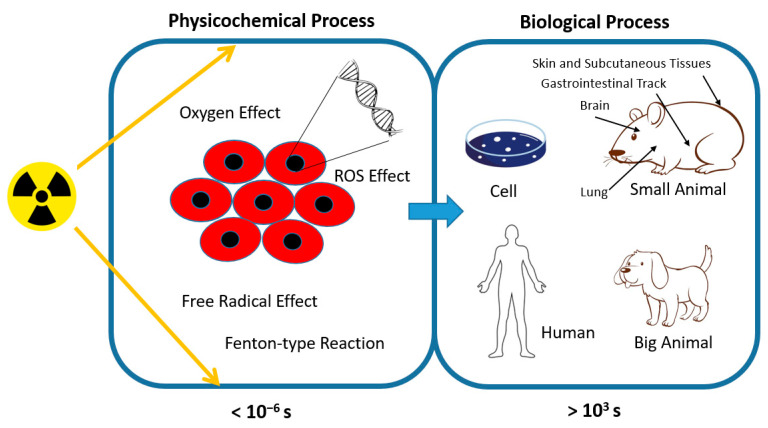
Summary of physicochemical and biological processes in FLASH-RT over time following irradiation.

However, the mechanism behind FLASH-RT and its effects on cellular function and response remain inadequately understood. Ongoing investigations are delving into factors such as oxygen depletion, free radical recombination, and the metabolism of peroxidized compounds to elucidate these complexities [13]. Hypotheses surrounding the oxygen depletion effect and reactive oxygen species (ROS)-mediated cell damage have been proposed. Figure 3 presents a summary of the physicochemical and biological processes within FLASH-RT across various time scales post-irradiation.

#### 2.1.1. Oxygen Depletion Effect

The oxygen depletion effect involves the rapid consumption of oxygen within cells during ultra-high-dose, short-duration radiation. This quick oxygen depletion induces transient radiation-induced hypoxia, influencing the differential responses observed between healthy and tumor cells in FLASH-RT [40]. The differential responses observed between healthy and tumor cells in vivo following FLASH-RT can be attributed to multiple hypotheses. One hypothesis suggests that the distinct types of DNA damage induced by FLASH-RT and conventional dose-rate irradiation contribute to the disparate responses of healthy and tumor cells [14]. Another perspective posits that solid tumors, often characterized by hypoxia, are less shielded from the transient hypoxia caused by FLASH-RT compared to healthy tissues, resulting in varied effects [41]. Furthermore, differences in the ability of cancer cells and normal cells to scavenge hydrogen peroxide products may contribute to the observed variations [42]. FLASH-RT rapidly depletes local oxygen in cells, generating hydrogen peroxide products. Healthy cells, with lower oxidant loads and higher catalase reduction reserves, may efficiently eliminate these products compared to tumor cells. Despite these theories, the precise mechanism underlying the differential responses remains unclear, necessitating further experimental validation of the various hypotheses.

Research indicates that, under low physiological oxygen conditions, many normal tissues can maintain cell populations for renewal and regeneration. UHDR radiation in FLASH-RT leads to rapid oxygen depletion, mimicking hypoxia and increasing normal cell radiation resistance [43,44]. This effect is particularly significant in oxygenated normal cells surrounding hypoxic tumors, as observed in CONV-RT. Water molecules in cells break down during UHDR radiation, generating ROS that indirectly damage DNA.

Recent studies challenge the widely accepted oxygen consumption mechanism of FLASH-RT. Growing evidence refutes the idea that oxygen depletion alone explains the protective effects of FLASH-RT on normal cells. Jansen et al. [45] found that, despite consuming more oxygen than CONV-RT, FLASH-RT did not deplete all oxygen in pure water, even at a 10 Gy total dose. This challenges the belief that complete oxygen consumption is the basis for the neural function preservation seen in mice. Epp et al. [46] and Adrian et al. [47] observed FLASH-RT’s protective effects only under very low oxygen concentrations, questioning the assumption of complete oxygen consumption. Adrian et al. [48] observed protective effects of FLASH-RT in normoxic conditions and oxygen-rich tissues like the lung. Furthermore, the oxygen depletion hypothesis fails to explain the similar antitumor effects of FLASH-RT and CONV-RT, as FLASH-RT may induce tumor cell resistance due to the inherently hypoxic nature of tumor tissues [49]. It is seen that the relationship between FLASH irradiation, oxygen consumption, and its impact on radiosensitivity is a subject of ongoing investigation, challenging traditional hypotheses on oxygen’s role in FLASH effect [12,43].

#### 2.1.2. ROS and Free Radical Effect

The UHDR delivered from FLASH-RT induces the generation of ROS and free radicals, crucial in elucidating the observed benefits. ROS, including superoxide anion (O_2_•−), hydrogen peroxide (H_2_O_2_), and hydroxyl radical (•OH), arise from the radiolysis of water molecules by the ionizing radiation used in FLASH irradiation [50]. These ROS engage in reactions with cellular components, such as DNA and proteins, causing oxidative stress and cellular damage [51]. Concurrently, the rapid release of high-energy radiation during FLASH-RT generates free radicals, like oxygen-centered (e.g., hydroxyl radicals) and carbon-centered radicals. These free radicals initiate chain reactions, contributing to oxidative damage in cellular components [52]. This mechanism may explain the advantages of FLASH-RT, such as the differential response between normal and cancerous cells to UHDRs. Normal cells, equipped with robust antioxidant systems, may better manage the increased oxidative stress induced by FLASH irradiation, while the rapid radiation delivery limits ROS and free radical diffusion, confining effects and sparing surrounding healthy cells [53].

Several recent studies have delved into the interplay of ROS and free radicals in FLASH-RT, regarding the underlying mechanisms and potential implications. One study involving zebrafish embryos exposed to conventional and FLASH-RT revealed minimal morphological effects, linking the enhanced radiation resistance of normal cells to decreased ROS levels [54]. Molecular dynamics simulations by Abolfath et al. [44] explored ROS generation near DNA, highlighting the formation of stable molecular states with limited diffusivity and lower potential for biological damage. Favaudon et al. [16] introduced the Transient Oxygen Depletion hypothesis, suggesting that preservation of FLASH-RT of normal cells is due to transient hypoxic radiation protection. However, conflicting findings on oxygen consumption during FLASH irradiation challenge this hypothesis. Montay-Gruel et al. [55] demonstrated that UHDR radiation inhibits ROS production through oxygen consumption, contributing to normal cell protection. Spitz et al. [56] emphasized differences in redox chemistry and iron content between normal and tumor cells, influencing the reaction of ROS and reducing cellular damage during FLASH-RT. Moreover, studies by Abolfath et al. [44], Labarbe et al. [32], and Lai et al. [57] explored the correlation between FLASH effect and oxygen concentration, ROS production rates, and the potential role of free radical recombination in cell protection. Blain et al. [52] observed a significant reduction in H_2_O_2_ production in FLASH-RT compared to CONV-RT in vitro. However, further investigations are needed to compare the differences in other ROS between FLASH-RT and CONV-RT. Overall, these findings collectively contribute to our understanding of the complex relationship between ROS, free radicals, and the observed effects of FLASH-RT on cells.

#### 2.1.3. Other Physicochemical Processes

Apart from the main oxygen and free radical recombination effect, there is another hypothesis regarding the Fenton-type reaction and peroxidized compounds in FLASH-RT suggested by Spitz et al. [56,58]. They found that the distinctive effect in FLASH-RT can be attributed to several interconnected factors. The UHDR characteristic of FLASH led to the rapid consumption of local tissue oxygen, resulting in the formation of reactive organic hydroperoxides. Importantly, Fenton-type reactions, which involve iron and contribute to cellular damage, are anticipated to be limited in normal cells compared to cancer cells due to lower levels of labile iron [59]. Given this, normal cells are expected to exhibit a more efficient removal of organic hydroperoxides compared to tumor cells. The differential ability to eliminate these reactive compounds becomes crucial, as tumor cells may struggle to remove hydroperoxides effectively [60]. Consequently, both FLASH and conventional dose rate irradiation are more isoefficient at killing tumor cells compared to normal cells, highlighting the potential of FLASH RT to selectively target cancerous cells while minimizing damage to normal surrounding structures.

### 2.2. Biological Process on Cell Killing

The biological process followed by the physicochemical process included the immune and inflammatory response, reduction in stem cell senescence, and vascular injury. They are demonstrated in various cell and preclinical experiments regarding different sites such as the brain, lung, gastrointestinal tract, and skin.

The immune and inflammatory hypothesis in FLASH effect proposed that the unique characteristics of FLASH, such as UHDR and the absence of an inflammatory response, can modulate immune and inflammatory processes in the tumor microenvironment, potentially enhancing antitumor effects [61,62,63]. Transforming growth factor-beta (TGF-β), a crucial proinflammatory cytokine, plays a specific role in the modulation of FLASH-RT effects compared to CONV-RT [64,65]. Studies have linked downregulated TGF-β signaling to radiation resistance in tumor-infiltrating T cells [66,67]. FLASH irradiation, characterized by reduced treatment time, allows more circulating immune cells to survive than CONV-RT, although this reduction in time may compromise the efficacy of fractionated irradiation [68]. FLASH radiation has been observed to induce a 1.8-fold increase in TGF-β levels 24 h post-irradiation, significantly lower than the 6.5-fold increase observed with conventional dose rates. This reduction in TGF-β levels suggests that FLASH radiation has the potential to minimize radiation-induced chronic inflammation [23,61]. Clinical studies support the idea that differences in high dose rate and total treatment time in FLASH-RT can preserve the immune system [69,70]. However, further research is needed to confirm the specific effects of FLASH exposure on chromosomal aberrations in circulating lymphocytes and proinflammatory cytokine levels in different cells compared to conventional dose-rate irradiation [69].

The protective effect of FLASH-RT in reducing stem cell senescence is pivotal, as senescent cells can release proinflammatory cytokines, potentially leading to pulmonary fibrosis and hindering cell regeneration post-radiation injury [13]. In a preclinical study by Fouillade et al. [71], mice irradiated under FLASH-RT demonstrated less lung injury and a comparable antitumor effect compared to conventional dose-rate irradiation. This protection may be linked to the preservation of stem cell replication ability, as the FLASH-RT group exhibited a 50% reduction in senescent stem cells. Notably, when stem cell senescence was induced in mice, the lung protection effect of FLASH-RT disappeared. Additionally, a study by Yang et al. [72] found that both tumor stem cells and normal tumor cells undergo apoptosis, scorch, and necrosis under FLASH-RT, with cancer stem cells showing stronger radiation resistance. However, further investigation is required to understand the impact of FLASH-RT on tumor stem cells compared to conventional dose-rate irradiation and its implications for the retention of antitumor effects. While the maintenance of stem cell division ability offers partial insights into the protective mechanism of FLASH-RT on normal cells, additional studies are essential to validate and explore other potential mechanisms, as well as to confirm experimental results [73].

RT-induced vascular injury is a significant component of radiation damage. Favaudon et al. [16] discovered that FLASH-RT reduces acute apoptosis of bronchial vessels compared to conventional dose-rate irradiation. In brain injury studies, FLASH-RT demonstrated superiority over conventional methods in preserving micro-vessel integrity, potentially benefiting cognitive function [55]. Nevertheless, existing evidence only establishes that FLASH-RT induces less vascular damage than conventional approaches, and the specific impact on the upstream gene regulatory pathway remains unclear. More detailed results of the above biological processes are explored in various cell and preclinical models.

#### 2.2.1. Cell and Preclinical Models in Brain and Lung

Extensive investigations into the biological processes of FLASH have focused on the brain, a late responding organ. Studies using 10 Gy whole brain irradiation revealed a dose-rate threshold of 100 Gy/s to trigger the FLASH effect, preserving neurogenesis and neuronal morphology while minimizing neuroinflammation [55,74]. Carbogen breathing during UHDR irradiation reversed the neuroprotection, demonstrating the influence of oxygen levels [41,44]. Further validations at lower dose rates and with a single fraction of 30 Gy confirmed neuroprotection, reducing reactive astrogliosis, microglial, and C3 complement activation. Moreover, vascular integrity and the blood–brain barrier were preserved following UHDR irradiation [75]. Evaluations in juvenile mice demonstrated spared memory loss and anxiety-like behaviors after whole brain irradiation at 8 Gy using UHDR [14]. Notably, UHDR spared normal brain tissue toxicity and reduced neuroinflammation, but its antitumor efficacy on glioblastoma was similar to conventional dose-rate irradiation [76]. These findings suggest that the antitumor efficacy of radiotherapy may be independent of dose rate, impacting neurocognitive decline in glioblastoma-bearing mice during fractionated regimens. On the other hand, Almeida et al. [77] investigated the antitumor immunological memory response in mice exposed to ablative doses of electron and proton beams, comparing conventional and FLASH dose rates. Their findings revealed that tumor responses remained largely independent of dose rate across various immunocompetent and immunodeficient mouse models. This observation challenges the notion of the immune response playing a significant role in the antitumor efficacy of FLASH-RT.

For preclinical experiment on lung, Favaudon et al. [16] pioneered the demonstration of the FLASH effect in the lung. Exposure to 17 Gy electron FLASH irradiation reduced delayed pulmonary fibrosis, contrasting with conventionally irradiated mice that developed extensive fibrotic lesions. This sparing effect correlated with reduced apoptosis in blood vessels and bronchi. At the tumor level, 15 Gy UHDR irradiation effectively controlled the growth of orthotopic TC-1 tumor cells in the lung, allowing feasible dose escalation up to 28 Gy for enhanced tumor control [14,78]. In the normal lung, studying cell repopulation after 17 Gy electron FLASH irradiation revealed minimized DNA damage and senescence in situ [79]. Further investigations in human fibroblast cell lines demonstrated reduced 53BP1 foci after 5.2 Gy FLASH irradiation compared to conventional, with RNA sequencing indicating attenuated fibrogenic and proinflammatory gene expression [71,80]. These findings suggest a genomic-level impact of FLASH-RT, influencing responses in both normal and tumor cells.

#### 2.2.2. Cell and Preclinical Models in Gastrointestinal Tract, Skin, and Subcutaneous Tissue

Preclinical results showed that FLASH irradiation offers advantages in acute responding organs, including the gastrointestinal tract and the hematopoietic system. In a FLASH electron irradiation with exposure equal to 14 Gy and dose rate equal to 216 Gy/s, intestinal function, epithelial integrity, and regenerating crypts are preserved while reducing DNA damage and apoptosis [81]. In a preclinical ovarian cancer model (ID8), FLASH irradiation demonstrates comparable antitumor efficacy to CONV-RT (0.08 Gy/s) [82]. Studies using spread-out Bragg peak irradiation [83,84] and pulsed synchrocyclotron [85] confirm enhanced survival with FLASH in pancreatic and gastrointestinal models. Importantly, UHDR reduces toxicity and improves crypt survival, making it a promising approach for minimizing gastrointestinal toxicity. These findings highlight the potential of FLASH-RT across different radiation modalities and emphasize its efficacy in controlling tumors while reducing normal tissue damage [86].

In a preclinical subcutaneous Lewis lung carcinoma model, exposure to 15 Gy electron at UHDR of 352 Gy/s preserved normal vasculature, while critical vascular collapse occurred with dose rate of 0.06 Gy/s in conventional irradiation [87]. The preservation of vasculature in FLASH irradiation was linked to reduced phosphorylation of myosin light chain (p-MLC), influencing endothelial cell contraction and immune cell infiltration. Studies using MLC kinase inhibitor (ML-7) in combination with CONV-RT replicated FLASH results, identifying the MLC pathway as a potential molecular target [87,88]. Dose escalation studies revealed reduced skin ulceration at 30 and 40 Gy in FLASH electron irradiation at a dose rate of 180 Gy/s compared to 0.07 Gy/s in CONV-RT. Proton beam studies confirmed similar results with 35 Gy delivered through FLASH scanning proton pencil beam and transmission proton beam [89]. These studies demonstrated decreased skin toxicity and leg contraction after FLASH irradiation, with comparable tumor control to CONV-RT in immunocompetent mice. In addition, FLASH proton RT with dose rate of 69–124 Gy/s spared skin, leg, and mesenchymal tissues from severe toxicities, while conventional proton RT (dose rate = 0.39–0.65 Gy/s) increased TGF-b1 levels in murine and canine skin. Both FLASH and conventional proton RT equally controlled subcutaneous and intramuscular sarcoma tumors [90]. Rudigkeit et al. [91] studied the proton-FLASH effect using an in vivo mouse ear model. They found that, in the 23 Gy group, no inflammation differences were noted. In the 33 Gy group, a dose rate of 9.3 Gy/s reduced ear swelling and inflammation scores by (57 ± 12)% and (67 ± 17)% and a dose rate of 930 Gy/s by (40 ± 13)% and (50 ± 17)% compared to conventional dose rate (0.06 Gy/s). Blood cytokines remained unchanged but estimated irradiated blood volume was 100 times higher with conventional than with FLASH, suggesting a role of blood in FLASH effect.

#### 2.2.3. Biological Models in Big Animal and Human

For big animal study, Vozenin et al. [92] identified 34 Gy as a tolerated and effective dose in FLASH-RT for cat-cancer patients. Phase III validation is underway. Dog patients with superficial solid tumors underwent dose escalation trials, with minimal follow-up, while a feasibility study in dogs with osteosarcoma showed minimal TGF-b production after 12 Gy FLASH protons [93,94]. Human clinical trials are limited at present [95,96], but a study on T-acute lymphoblastic leukemia patient-derived xenografts revealed sensitivity to UHDR [97], suggesting a gene-related susceptibility profile. Positive and negative FLASH effect studies are summarized [90], highlighting that UHDR and conventional dose rate irradiation are similarly effective in tumor control, emphasizing tumor sensitivity independence of dose rate. A hypothesis from Spitz et al. [56] suggests that differential distribution of organic hydroperoxides after FLASH vs. conventional irradiation contributes to the therapeutic index, with antioxidants effectively removing hydroperoxides in normal cells but not in tumors.

## 3. Future Prospective

FLASH-RT as an innovative tumor treatment has captivated the attention of the radiation oncology community. This review delves into current hypotheses explaining the underlying physicochemical and biological processes, encompassing both theoretical and experimental aspects of the FLASH effect. Despite recent advancements, significant hurdles hinder the clinical translation of FLASH-RT, partially due to the inadequate understanding of cell killing under the UHDR radiation beams. Enhancing cell and preclinical experiments requires crucial technical advancements, including the development of a delivery system capable of simultaneously administering multiple FLASH irradiation beams [98,99]. Real-time adaptation and understanding the intricate mechanisms of the FLASH effect pose additional challenges [100,101].

While studies have illuminated early effects of FLASH-RT, its late and overall effects remain unknown [96,102]. Verification of scientific findings and controlled FLASH-RT vs. CONV-RT studies are vital for future research. Moreover, the lack of a comprehensive simulation platform for FLASH irradiation necessitates the development of advanced tools to accelerate understanding [103]. The future focus of the radiation oncology community is decoding the FLASH effect’s mechanism and clinical feasibility.

Further animal experiments are essential to demonstrate the FLASH protective effect on healthy cells. Standardization of experimental conditions, including radiation sources and field shapes, is crucial for accurate comparisons. Various modified irradiation systems, such as electron linear accelerators, synchrotron light sources, and proton accelerators, offer promise but require refinement for broader clinical application [104,105].

To progress, clinical confirmation of the FLASH effect in cancer patients, redefinition of irradiation doses, and addressing urgent challenges in clinical transformation are imperative [95]. Lack of clinical data, uncertainty about tumor metastasis, unclear long-term effects, limited equipment, and questions regarding treatment planning systems must be addressed for the successful integration of FLASH-RT into mainstream radiotherapy practices [20,25,106]. The potential benefits of FLASH-RT based on expectations and current knowledge [107] make it a promising avenue for the future of cancer treatment [10]. However, extensive research, clinical trials, and technological advancements are essential to overcome current limitations and ensure its safe and effective application.

## 4. Conclusions

In conclusion, our review illuminates the intricate mechanisms that unlock the cell-killing potential of FLASH-RT. The combined impact of physicochemical and biological factors is pivotal to FLASH-RT in eliminating cancer cells while protecting the normal cells. The physicochemical process, including the role of reactive ROS and radiolysis, have been postulated as key contributors to the enhanced cell-killing effect in FLASH-RT. However, a deeper understanding of these processes is imperative for a comprehensive grasp of the underlying biological responses. The emerging insights into FLASH-RT-related radiobiological processes open avenues for future research aimed at elucidating the intricate interplay between radiation and cellular responses. Biological processes, particularly the protective effect observed in healthy cells, pose challenges and opportunities for clinical translation. The need to distinguish between the effects induced by FLASH and conventional irradiation underscores the importance of unraveling the long-term and overall impacts of FLASH-RT on biological systems. Collaboration across disciplines and sustained efforts in research and clinical applications will be pivotal. The ongoing pursuit of understanding the mechanisms involved in the cell-killing efficacy of FLASH-RT paves the way for transformative advancements in the field of radiation oncology. As FLASH-RT continues to advance at top speed due to clinical needs as a promising cancer treatment modality, our review underscores the ongoing necessity for further research to elucidate its mechanisms comprehensively. There are current limitations in clinical experimentation due to incomplete understanding. So, this review showing the growing body of supportive evidence from cell and preclinical studies suggests promising prospects for the safe implementation of FLASH-RT in human patients. Moving forward, bridging the gap between preclinical validation and clinical application will be pivotal in realizing the full potential of FLASH-RT as an effective and safe treatment option for cancer patients.

## Figures and Tables

**Table 1:** Comparison of FLASH-RT and CONV-RT.

Aspect	FLASH-RT	CONV-RT
Treatment Time	Ultra-fast (milliseconds)	Typically seconds to minutes
Dose Rate	Extremely high (>40 Gy/s)	Moderate to high (0.001–0.4 Gy/s)
Normal Cell Sparing	Enhanced due to UHDR	Limited, increased risk to normal cells
Oxygen Effect	Reduced due to ultra-short exposure	Present, potential impact on tumor response
Radiobiological Effect	Increased therapeutic index	Standard radiobiological principles
Fractionation	Single or few fractions possible	Multiple fractions common
Patient Comfort	Reduced overall treatment time	Longer treatment sessions
Machine Wear and Tear	Potentially reduced	Standard wear and tear
Integration with Imaging	Compatibility with advanced imaging	Standard imaging requirements
Organ Motion during Treatment	Reduced impact due to faster delivery if the tumor position is known immediately prior to treatment	Continuous monitoring and adaptation
Patient Throughput	Potentially increased	Treatment duration may impact throughput
Clinical Trial Status	Investigational, ongoing research	Established, widely practiced
Cost and Accessibility	Potential for higher costs	Generally more accessible
